# Motile and Chemotactic Minicells and Minicell-Driven Biohybrids Engineered for Active Cargo Delivery

**DOI:** 10.1021/acsami.5c04638

**Published:** 2025-06-12

**Authors:** Irina Kalita, Remy Colin, Sarah Hoch, Saadet Fatma Baltaci, Metin Sitti, Victor Sourjik

**Affiliations:** † Max Planck Institute for Terrestrial Microbiology and Center for Synthetic Microbiology (SYNMIKRO), Marburg 35043, Germany; ‡ Physical Intelligence Department, Max Planck Institute for Intelligent Systems, Stuttgart 70569, Germany; § Stuttgart Center for Simulation Science, University of Stuttgart, Stuttgart 70569, Germany; ∥ School of Medicine and College of Engineering, Koç University, Istanbul 34450, Turkey

**Keywords:** bacterial minicells, microswimmers, motility, chemotaxis, active biohybrids, drug delivery

## Abstract

Bacterial minicells are submicrometer-sized spherical compartments produced by bacteria as a result of aberrant cell division. Minicells have a similar cellular composition to the parental bacteria but lack chromosomal DNA and are thus unable to proliferate. Due to that, minicells have attracted attention as potential means of effector delivery in bioengineering and biomedical applications. However, until now, the efficiency of delivery by minicells has been limited by passive collisions with their targets. To develop minicell-based active delivery, here we engineer Escherichia coli strains generating motile minicells with enhanced swimming properties by introducing genetic modifications specifically targeting flagella number, length, and rotation speed. The engineered minicells preserve motility over an extended period of time and, in contrast to parental E. coli cells, increase their swimming speed for the intermediate viscosity of the medium. Despite their small size, minicells show an efficient chemotactic response and utilize the same chemotactic strategy as parental E. coli cells. Moreover, we develop a procedure for conjugating minicells with cargo particles and demonstrate that such minicell-driven biohybrid swimmers are chemotactic and thus capable of actively accumulating at the source of an attractant. These engineered chemotactic minicells and minicell-based biohybrids can serve as cargo delivery platforms with active targeting, thus overcoming the challenges posed by nontargeted therapies.

## Graphical abstract

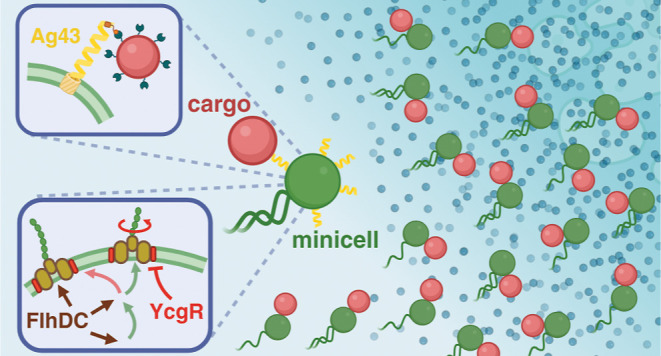

## Introduction

Due to their adaptability to complex microenvironments and ease of microbial genome manipulation, bacteria have high potential for biomedical applications,^1^ including probiotics design,^2^ targeted drug delivery,^3–5^ biosensing,^6^ and bioimaging.^7^ Recent advances in genetic engineering and synthetic materials have opened new possibilities for integrating the natural properties of bacteria with the enhanced functionalities of artificial biomaterials.^8–10^ These rationally designed bacterial hybrids are able to accomplish customized tasks that neither component could achieve independently, such as remote control and precise steering or activation through magnetic fields,^11,12^ near-infrared light,^13,14^ and ultrasound waves.^15^ Despite the progress achieved by bacteria-based drug delivery platforms, further advancements are still needed to enable autonomous navigation, precise localization, and efficient penetration into target areas.^16^ Engineered biohybrids that exploit the self-propelling and sensing abilities of motile bacteria offer a compelling approach to address these challenges.^11,17^

In fluid environments, most bacteria, including Escherichia coli, are capable of efficient active self-propulsion that is achieved by the rotation of flagellar filaments, powered by the electrochemical potential across the cell membrane.^18^ Bacteria alternate persistent swimming in a specific direction (runs) and random changes in direction (tumbles) to explore their environment. While swimming, they compare chemical concentrations and adjust their motion by extending runs toward more favorable conditions.^19^ To tightly control production of costly flagellar filaments,^20^ the expression of flagellar and chemotaxis genes is regulated via a hierarchical network.^21^ The master regulator FlhD/FlhC activates transcription of the early genes encoding components of the hook-basal body as well as of the transcriptional regulator of late flagellar genes FliA (σ^28^) and its inhibitor FlgM (anti-σ^28^). FlgM is secreted once the basal body of the flagellum is assembled, enabling transcription of the late genes, which encode motor torque-generator subunits, chemotaxis proteins, and flagellin FliC, to be activated by FliA. Flagellar rotation is further tuned by the motor brake protein YcgR that is responsive to the levels of the bacterial second messenger cyclic di-GMP (c-di-GMP).^22–25^

While the prospects of harnessing bacterial locomotion and sensing in biomedical applications have become increasingly recognized, the unpredictability of proliferation and genetic mutations of replicating bacteria within the host poses serious biosafety concerns.^8^ In this regard, the chromosome-less, nondividing bacterial products, known as minicells, present a clear advantage over reproducing bacteria. Deletion of the genes regulating positioning of the cell division site (the *minCDE* operon in E. coli) perturbs the standard midcell division, leading to overall elongation of parental cells and misplaced positioning of division sites,^26^ with divisions close to a cell pole leading to production of submicron chromosome-less minicells.^27^ Apart from lacking the chromosomal DNA, minicells retain structural and functional components of the parental cells and can execute most of cellular processes, including plasmid replication,^28,29^ mRNA and protein production,^30,31^ and ATP synthesis.^32^ Minicells have been used not only in a wide spectrum of fundamental research^26,33–35^ but also as achromosomal drug delivery vectors.^36^ Packaged with a chemotherapeutic drug and decorated with antibodies to cell-surface tumor-specific receptors, bacterial minicells were shown to accumulate in tumor xenograft models and endogenous tumors.^37–42^ While being further supported by phase I clinical trials,^43–45^ minicell-based approaches are hampered by off-target effects and drug leakage. Using motile and chemotactic minicells and attaching cargo externally may allow reduction of these limitations, enhancing the specificity and efficiency of targeted delivery.

To establish an active minicell-driven delivery platform, herein we engineered bacterial minicells and minicell-based biohybrids with enhanced swimming and chemotactic properties, by combining several genetic modifications that increase the flagella number and rotation speed. We further established a procedure for the efficient purification of motile minicells. Despite their much-reduced size, these engineered motile minicells reach a high average swimming speed and qualitatively maintain the chemotactic behavior of parental E. coli cells. We further demonstrated that the engineered minicells preserve motility for over 4 days and, in contrast to the normal-sized E. coli cells, increase their swimming speed for intermediate viscosity of the medium. Furthermore, by modifying the surface of minicells, we developed a procedure to fabricate motile minicell-based biohybrids that are capable of active accumulation at a source of a chemoattractant.

## Results and Discussion

### Production and Purification of Motile Minicells

To establish a procedure for efficient purification of motile minicells (Figure 1A) produced by the E. coli MG1655 Δ*minCDE* strain or its derivatives (Table S1), several modifications have been introduced into the previous minicell separation protocols.^46–48^ First, to allow for greater minicell accumulation in a cell culture, we extended the exponential phase of growth and harvested the cultures at the early stationary phase (OD_600_ = 0.8–1.0). The collected culture of the E. coli Δ*min* strain predominantly consisted of normal-sized and elongated cells (Figure 1B, left) with an underrepresented subpopulation of minicells (Figure 1C, left panel; minicell subpopulation is highlighted). Notably, Δ*min* cells exhibited not only a broader cell size distribution but also a lower average swimming speed compared to the wild type, suggesting that their motility is suboptimal. Second, after the first centrifugation that removed a fraction of parental cells (Figure 1B,C, middle), the remaining parental cells were eliminated by restarting the growth of the culture in a fresh medium containing ceftriaxone. This antibiotic inhibits bacterial cell wall synthesis,^48,49^ leading to the lysis of growing and dividing parental cells but not affecting nonreplicating minicells. The final purified suspension consisted nearly exclusively of minicells (Figure 1B,C, right), with an increasing concentration of ceftriaxone resulting in a higher purity of the minicell subpopulation (Figure S1). Importantly, the swimming of minicells remained unaffected by purification and ceftriaxone treatment (Figure 1D).

**Figure 1:**
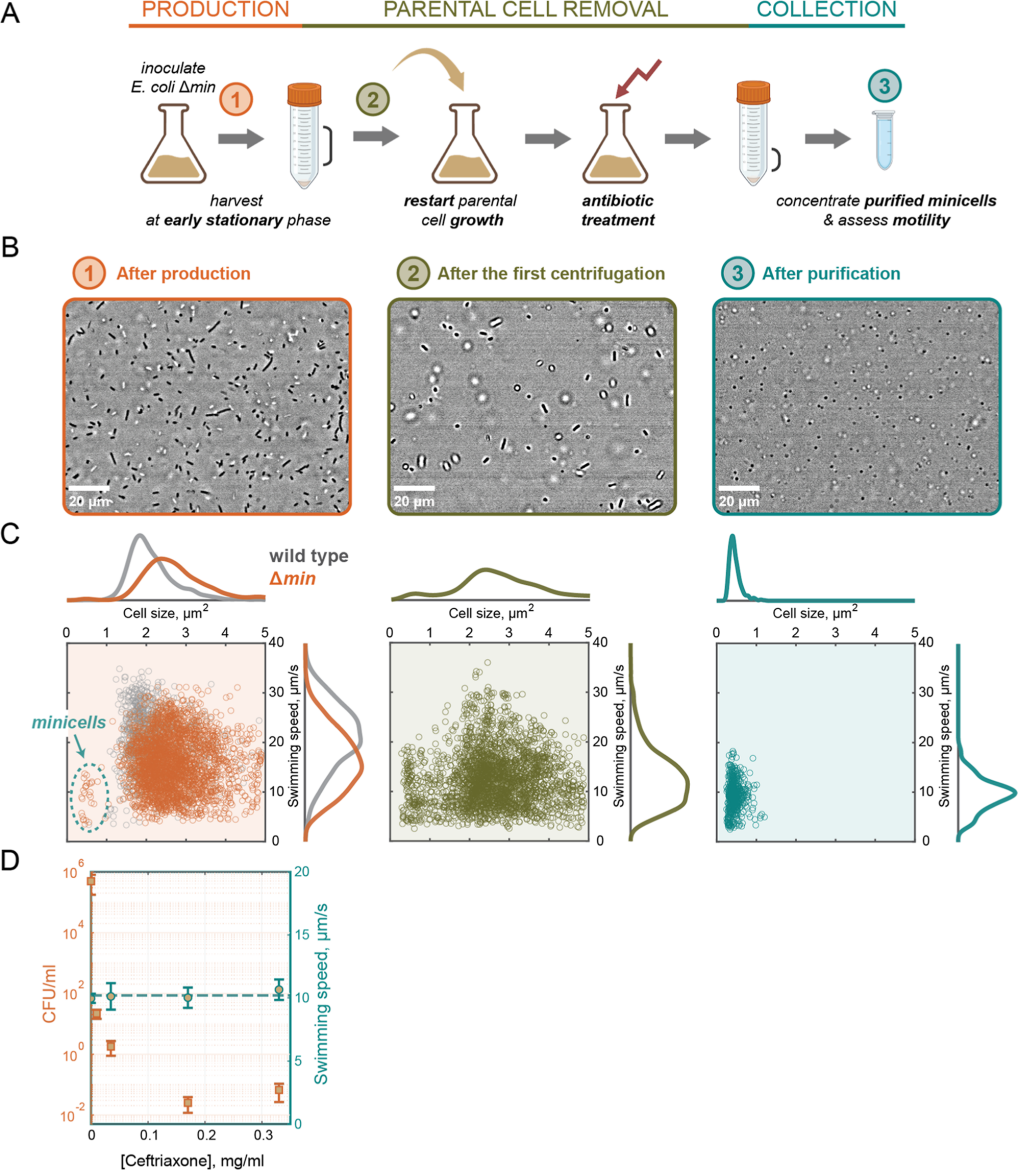
Protocol for highly efficient purification of motile bacterial minicells. (A) A schematic of the protocol for purification of motile minicells from a minicell-producing Escherichia coli strain. Three main stages of the protocol are highlighted: minicell production, parental cell removal, and final collection of the purified minicells. (B) Examples of bright-field images of an E. coli MG1655 Δ*min* cell suspension taken at each stage of the protocol: after production (left), after the first centrifugation (middle), and after purification with the described protocol (right). The scale bars are 20 μm. (C) Single-cell correlation between swimming speed and cell size for the corresponding stage of the protocol. The marginal distributions of swimming speed and cell size are plotted on the sides. In the left panel: normal-sized wild-type cells are shown (in gray) for a relative comparison and a subpopulation of minicells is highlighted. (D) Colony-forming units (CFU) per ml of the initial culture taken for the purification (left *y*-axis) and swimming speed of minicells (right *y*-axis) as functions of the ceftriaxone concentration used during the purification process. For CFU quantification, 20 mL of the initial culture was taken to purify and concentrate minicells in the final volume of 100 μL, which was then plated onto Luria broth (LB) plates for colony counting. The average values are calculated as the mean among at least three replicated experiments for each antibiotic concentration; the error bars represent standard deviations. The dashed line indicates the average swimming speed of minicells calculated for all experiments.

To further confirm that our purification procedure can essentially eliminate viable parental cells, we performed counting of colony-forming units (CFUs) upon plating the purified minicell suspension onto agar plates (Figure 1D). The number of viable parental cells indeed decreased by 7 orders of magnitude when ceftriaxone was applied for 1 h at the concentration of 170 μg/mL or higher, which corresponded to one viable parental cell remaining in the suspension obtained from 40 mL of the initial cell culture. We therefore used an intermediate concentration of 250 μg/mL in all experiments. By avoiding ultracentrifugation and filtration, minimizing centrifugation speeds and pipetting, and applying ceftriaxone treatment, we thus established a procedure for separation of motile minicells with high purification efficiency (Movie S1).

### Genetic Engineering of Flagellar Regulation Improves Minicell Motility

In comparison to that of wild-type cells, the swimming speed of minicells was found to be twice slower (Figure 1C). Aiming to enhance minicell motility, we applied a genetic engineering approach to tune the rotation speed, flagellar length, and the overall production of flagellar and chemotaxis proteins (Figure 2A). To increase the flagellar rotation speed, we deleted the gene encoding the protein YcgR. YcgR is known to function as a flagellar brake by interacting with the stator protein MotA and the switch-complex proteins FliG and FliM in a c-di-GMP-dependent manner, which leads to reduced torque and rotational speed of the motor and, consequently, decreased swimming speed of E. coli.^22,23,25,50^ Since minicells are derived from parental E. coli cells, we anticipated that the YcgR-mediated mechanism regulating flagellar motor function would be preserved in minicells. In order to elevate the expression of all flagellar genes, including those coding for the structural components of the basal body, hook, flagellar filaments, and chemotaxis proteins, we overexpressed the master regulator FlhD/FlhC (FlhDC) from the *flhDC* expression plasmid. The transcriptional factor FlhD/FlhC is known to activate a hierarchical network controlling the expression of all flagellar and chemotaxis genes^21,51^ and its expression above the wild-type level has been reported to increase the flagellar number and length in wild-type E. coli cells.^52^ Lastly, to specifically target flagellar length, we removed the inhibitor FlgM.^20,51^

**Figure 2:**
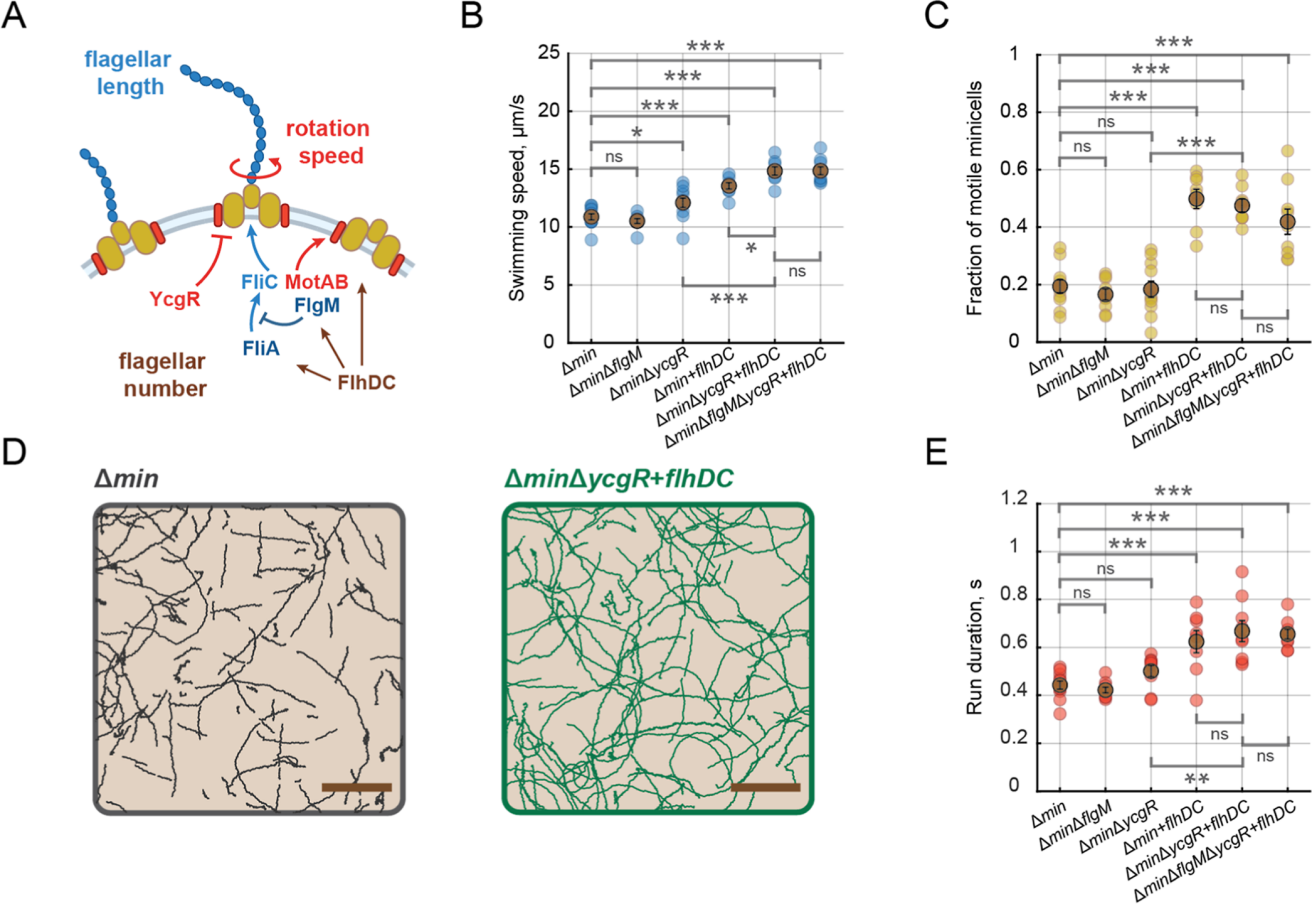
Genetic engineering of flagellar regulation improves minicell motility. (A) A simplified schematic showing the regulation of flagellar gene expression and motor rotation in E. coli. See the text for details. (B) Swimming speed of minicells generated by respective genetically modified strains. Swimming speed was calculated for the motile minicells only. (C) A fraction of the motile minicells produced by indicated strains. (D) Examples of trajectories of individual minicells detected with single cell tracking for the minicells produced by Δ*min* (left) or Δ*min*Δ*ycgR* + *flhDC* (right) strains. 100 randomly chosen trajectories have been visualized for each sample. The scale bars are 20 μm. (E) Average duration of the runs computed with particle tracking analysis for minicells generated by indicated modified strains. In (B,C,E), each data point represents the average swimming speed of one population of purified minicells, while dark points show the mean among the averages of the replicated experiments (at least six for each strain). The error bars represent the standard error of the mean. A two-sample *t*-test was used to compare different strains and calculate significance values: *P* ≤ 0.05 (*), *P* ≤ 0.01 (**), *P* ≤ 0.001 (***), *P* > 0.5 (ns).

To investigate the effects of these modifications on minicell motility, we first introduced each of them individually into the minicell-producing Δ*min* strain. The swimming speed and fraction of motile minicells produced by the respective E. coli strains were quantified using single-cell tracking and averaged across purified populations containing hundreds of minicells. Both *ycgR* deletion and FlhDC overexpression resulted in a significant enhancement of minicell motility. Whereas minicells derived from the strain overexpressing FlhDC exhibited both higher swimming speed and the fraction of motile minicells, minicells lacking the flagellar brake protein YcgR demonstrated faster swimming without the increase of the motile subpopulation (Figures 2B,C and S2). A combination of *ycgR* deletion and FlhDC overexpression resulted in an additive effect, altogether resulting in a significant improvement of the swimming speed compared to the original Δ*min* strain or minicells that only overexpress FlhDC.^47^ In contrast, *flgM* deletion did not lead to any significant increase in minicell swimming, either alone or in the combination with Δ*ycgR* and FlhDC overexpression.

We therefore subsequently used E. coli Δ*min*Δ*ycgR* + *flhDC* for the production of highly motile minicells. Besides their increased swimming speed, minicells generated by this strain exhibited more persistent trajectories compared to the original Δ*min* strain (Figure 2D and Movie S2). Quantification of the durations of runs, defined as segments of trajectories between tumbles, showed that the average run duration followed a trend similar to the swimming speed, with the longest run durations observed for Δ*min*Δ*ycgR* + *flhDC* minicells (Figure 2E).

To investigate the underlying causes of the swimming speed improvement in Δ*min*Δ*ycgR* + *flhDC* minicells, we determined the number and rotation speed of flagellar filaments using flagellar staining^53^ (Figure 3A). In agreement with our expectations, the Δ*min*Δ*ycgR* + *flhDC* strain produced a higher fraction of flagellated minicells that carry more than one flagellum (∼41%) compared to the initial Δ*min* strain (∼11%) (Figure 3B). While an exact quantification of filaments above two was complicated by flagellar bundling, examples with more than four flagella were seen for Δ*min*Δ*ycgR* + *flhDC* minicells but never appeared in Δ*min* minicells (Figure 3A). Although the identification of nonflagellated minicells in these images was limited due to their small size and lack of distinctive features, the higher fraction of motile Δ*min*Δ*ycgR* + *flhDC* minicells (Figure 2C) suggests that this strain also produces more flagellated minicells.

**Figure 3:**
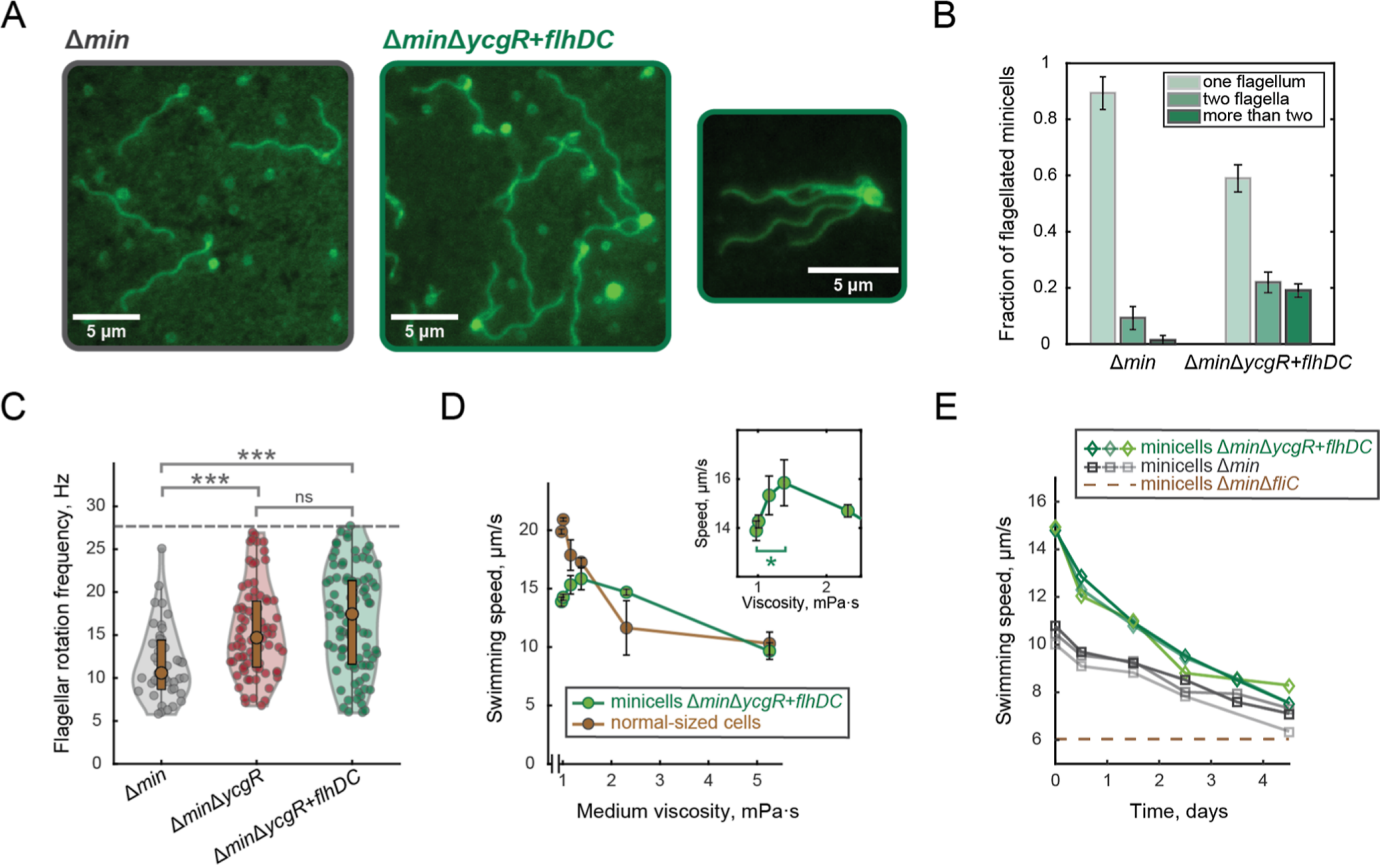
Characterization of enhanced motility of engineered minicells. (A) Examples of wide-field fluorescence images of minicells, produced by Δ*min* and Δ*min*Δ*ycgR* + *flhDC* strains, labeled with Alexa Fluor 594 carboxylic acid succinimidyl ester dye and immobilized onto 2% agar pads. An example of a minicell, generated by the Δ*min*Δ*ycgR* + *flhDC* strain, with more than four flagella, is shown in the right panel. The scale bars are 5 μm. (B) Quantification of the fraction of flagellated minicells produced by Δ*min* and Δ*min*Δ*ycgR* + *flhDC* strains. The numbers of minicells with one flagellum, two flagella, and more than two flagella were normalized to the total number of flagellated minicells. Total number of flagellated minicells included in the quantification: 673 (Δ*min*) and 613 (Δ*min*Δ*ycgR* + *flhDC*). The error bars represent the standard deviation among three labeling experiments. (C) Violin plots showing the distributions of flagellar rotation frequency identified in the power spectral density (PSD) profiles for single minicells (examples of PSDs are shown in Figure S3). Total number of minicells included in the analysis: 61 (Δ*min*), 107 (Δ*min*Δ*ycgR*), and 107 (Δ*min*Δ*ycgR* + *flhDC*). A two-sample *t*-test was used to compare different strains and calculate significance values: *P* ≤ 0.05 (*), *P* ≤ 0.01 (**), *P* ≤ 0.001 (***), *P* > 0.5 (ns). (D) Minicell swimming speed measured as a function of medium viscosity for Δ*min*Δ*ycgR* + *flhDC* minicells and normal-sized wild-type cells. The viscosity of the medium was modified with Ficoll 400 in the range between 0 and 10% (w/v) Ficoll (corresponding to 1–5.3 mPa·s). The calibration function between the percentage of Ficoll and medium viscosity, obtained by fitting the published data^69^ (Figure S4a). The data points in the graph were calculated as the average between three replicated experiments for wild-type and four replicates for minicells; the error bars represent the standard error of the mean. The range of viscosities, within which the swimming speed increase has been observed, is shown in the inset. The significance of the maximal increase was calculated using a paired one-side *t*-test (*p* = 0.028). (E) Swimming speed of minicells produced by Δ*min* and Δ*min*Δ*ycgR* + *flhDC* strains measured in a time-course experiment over 4 days after the purification. During the time-course, minicells were kept at 18 °C in Tryptone broth (TB) daily supplemented with 1% glucose. Three experiments were performed for each strain. The horizontal dashed line indicates the lowest threshold for swimming velocity (measured for nonmotile minicells produced by the Δ*min*Δ*fliC* strain).

To additionally untangle the effect of *ycgR* knockout, we quantified the frequency of flagellar rotation. For that, we recorded time-lapse movies of swimming minicells with labeled flagella in a solution of 10% (w/v) Ficoll 400, with a viscosity 5 times that of water (5.3 mPa·s) to satisfy the time resolution of our setup (Movies S3 and S4). Power spectral densities of the fluorescence intensity, computed for individual labeled swimming minicells, displayed dominating frequency peaks corresponding to flagellar rotation (Figure S3, Experimental Methods). Consistent with the established role of YcgR as a flagellar brake protein that slows the rotation of the motor, Δ*ycgR* minicells showed a prominent increase in flagellar rotation frequency (15.5 Hz) compared to the original Δ*min* minicells (11.7 Hz) (Figure 3C). An additional minor increase was observed for Δ*min*Δ*ycgR* + *flhDC* minicells (16.8 Hz), possibly due to an increased expression of the flagellar motor stator units expected at higher levels of the FlhDC activator. Altogether, we concluded that motile minicells produced by the Δ*min*Δ*ycgR* + *flhDC* strain, on average, have (i) more flagella per minicell as a result of upregulation of all structural components of flagella and (ii) higher rotation speed. The latter effect is primarily due to the *ycgR* deletion, highlighting its importance for the performance of engineered motile minicells.

Since many natural environments, including animal body fluids, have higher viscosity than water, we investigated how the motile behavior of minicells depends on the medium viscosity in the intermediate range (0–5.3 mPa·s), adjusted using Ficoll (Figure S4a). Consistent with the literature,^54–56^ wild-type E. coli bacteria showed a monotonic decrease in their swimming speed when the environment became more viscous (Figure 3D). In contrast, the trend for minicells was fundamentally different, with the swimming speed significantly increasing for the intermediate viscosity to the maximum of ∼16 μm/s at 1.4 mPa·s (2% Ficoll). This viscosity value is close to the normal range of human blood.^57^ Consistently, minicell trajectory tracks recorded in 2% Ficoll appeared notably straighter in comparison to the ones in the buffer (Figure S4b).

Given the functional metabolic activity of minicells,^30,58,59^ we assessed how long they can maintain membrane potential to power flagellar rotation by measuring their swimming speed over several days of incubation at room temperature (18 °C) in the presence of glucose. Although the swimming speed decreased over time, minicells remained motile for more than 4 days after being purified from parental cells (Figure 3E). This observation confirms that minicells can generate enough energy to sustain the costly swimming behavior for an extended period of time.

### Engineered Minicells Maintain the Chemotactic Strategy of Wild-type Bacteria

We next aimed to examine whether the achieved improvement in minicell motility would be reflected in a more efficient chemotactic performance. Minicells generated by the Δ*min*Δ*ycgR* + *flhDC* strain exhibited not only faster swimming (Figure 2B) but also more persistent trajectories and longer runs than those produced by the original Δ*min* strain (Figure 2D,E), enabling more accurate temporal comparisons of concentration changes while swimming. According to established models of bacterial chemotaxis, this should lead to increased chemotactic drift velocity in the direction of an attractant gradient, particularly given the quadratic dependence of drift on swimming speed.^60–62^ To verify this expectation, we quantified the chemotaxis performance of minicells produced by different strains by tracking minicells in a microfluidic chamber with a gradient of the nonmetabolizable chemoattractant α-methyl-dl-aspartate (MeAsp) (Figure 4A). In this assay, a linear concentration gradient of the chemoeffector was shown to be established within 1 h and remain stable for several hours.^62,63^ As anticipated, both the average chemotactic drift and the chemotactic bias (drift normalized by velocity) were highest for Δ*min*Δ*ycgR* + *flhDC* minicells, with the chemotactic drift of ∼1 μm/s that is three times faster than minicells produced by the original Δ*min* strain and two times faster than minicells produced by the Δ*min* + *flhDC* strain^47^ (Figures 4B and S5a). The chemotactic efficiency, defined as chemotactic bias normalized by swimming speed,^62^ was also highest for the improved minicells derived from the Δ*min*Δ*ycgR* + *flhDC* strain (Figure S5b). This again highlights the important role of *ycgR* deletion in enhancing both the motility and chemotactic performance of minicells.

**Figure 4:**
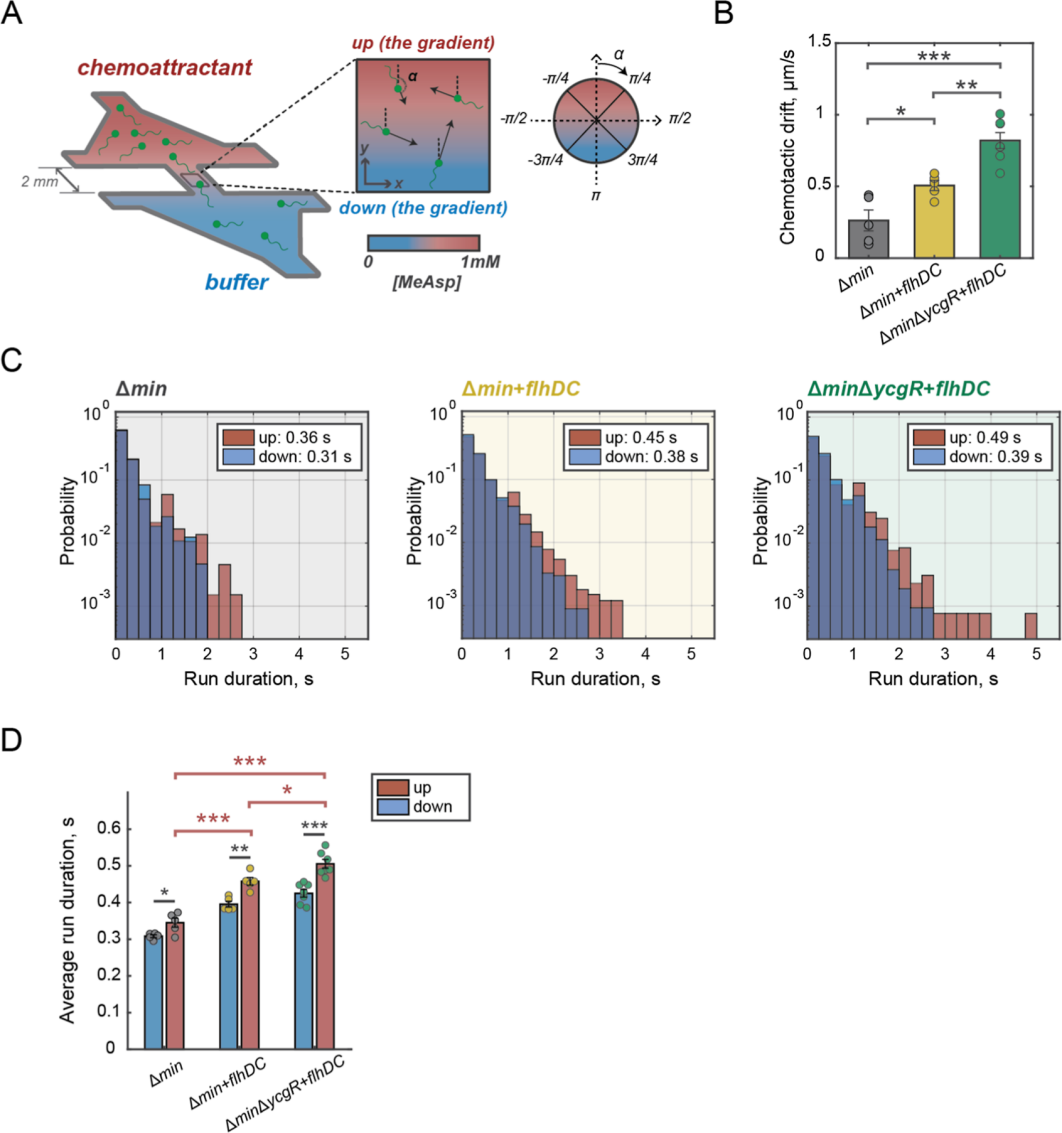
Engineered minicells exhibit enhanced chemotactic performance. (A) A schematic of the experimental setup of the microfluidic device for quantitative measurements of chemotactic response. Flagellated minicells are illustrated in green (shown not to the scale). Minicell movement is recorded in the middle of a microfluidic channel with an established linear gradient of a nonmetabolizable chemoattractant α-methyl-dl-aspartate (0–1 mM MeAsp). Single trajectories of minicells are tracked and the runs are separated based on their directionality: either up (|α| < π/4) or down (3π/4 < |α| < π) the attractant gradient. (B) Chemotactic drift measured for the minicells produced by Δ*min*, Δ*min* + *flhDC*, and Δ*min*Δ*ycgR* + *flhDC* strains in the presence of a 0–1 mM MeAsp gradient. Each data point represents the average chemotactic drift for a population of purified and tracked minicells, while the bars show the mean among the averages of the replicated experiments (at least five for each strain). The error bars are the standard errors of the mean. A two-sample *t*-test was used to calculate significance values: *P* ≤ 0.05 (*), *P* ≤ 0.01 (**), *P* ≤ 0.001 (***), *P* > 0.5 (ns). (C) Run duration distributions from a representative experiment in the presence of the MeAsp gradient (0–1 mM) for the minicells produced by Δ*min*, Δ*min* + *flhDC*, and Δ*min*Δ*ycgR* + *flhDC* strains. The runs shorter than 5 frames (0.1 s) were excluded from the analysis. All other runs were separated based on their directionality: either up or down the gradient (as shown in A). The average run durations in both directions are shown in the legends. The number of analyzed trajectories: 909 (Δ*min*), 5121 (Δ*min* + *flhDC*), and 2107 (Δ*min*Δ*ycgR* + *flhDC*). (D) Average run duration in the presence of the 0–1 mM MeAsp gradient for the minicells produced by Δ*min*, Δ*min* + *flhDC*, and Δ*min*Δ*ycgR* + *flhDC* strains. The runs were conditioned on their directionality—either up or down the gradient of MeAsp. The average values were calculated for at least four replicated experiments for each strain. The error bars are the standard errors of the mean. To compare run durations up and down (the gradient), a paired one-side *t*-test has been applied. A nonpaired *t*-test was used to compare run durations toward MeAsp for different strains. Significance marks stand for: *P* ≤ 0.05 (*), *P* ≤ 0.01 (**), *P* ≤ 0.001 (***), *P* > 0.5 (ns).

Elongation of runs up the attractant gradient is a well-established chemotaxis strategy of wild-type E. coli.^19^ To investigate whether minicells utilize a similar strategy, we analyzed the duration of runs up or down the chemoattractant gradient (Figure 4A). Minicells generated by all three strains extended their runs up the attractant gradient, with the largest difference between runs in the up and down directions observed for faster minicells (Figure 4C,D). Thus, although their directional bias remains below that of the wild-type E. coli (Figure S5a), minicells execute the conventional chemotaxis strategy of the wild-type cells, which primarily relies on extending runs up the attractant gradient (Figure S6). Remarkably, the chemotactic efficiency of the minicells, generated by the Δ*min*Δ*ycgR* + *flhDC* strain, is nearly equivalent to that of wild-type E. coli (Figure S5b), suggesting the high fidelity of their sensing capacity.

### Motile and Chemotactic Biohybrids of Engineered Minicells Exhibit Accumulation toward the Source of an Attractant

Finally, we sought to investigate whether the motility and chemotaxis of minicells could be harnessed for active cargo delivery. We applied a previously described labeling approach,^17^ where streptavidin-coated beads were attached to the cell surface via a biotinylated version of the autotransporter antigen 43 (Ag43) (Figure 5A). This approach exploits the exceptionally strong streptavidin–biotin interaction, which exhibits a dissociation half-life of several days under physiological conditions.^64^ Since Ag43 was shown to be distributed across the cell surface with a preference for the cell poles,^17,65^ we anticipated that it would be inherited by minicells from the parental cells. Indeed, using a fluorescent analogue of streptavidin (NeutrAvidin) for specific labeling, we verified that the biotinylated Ag43 localized at the surface of minicells (Figure S7). To demonstrate specific attachment via biotin–streptavidin interaction, we conjugated minicells expressing green fluorescent protein (GFP) with 1.4 μm red fluorescent streptavidin-coated microparticles. Colocalization of minicells with microparticles was indeed frequently observed (Figure 5B), and it could be additionally confirmed by using scanning electron microscopy (Figure 5C).

**Figure 5:**
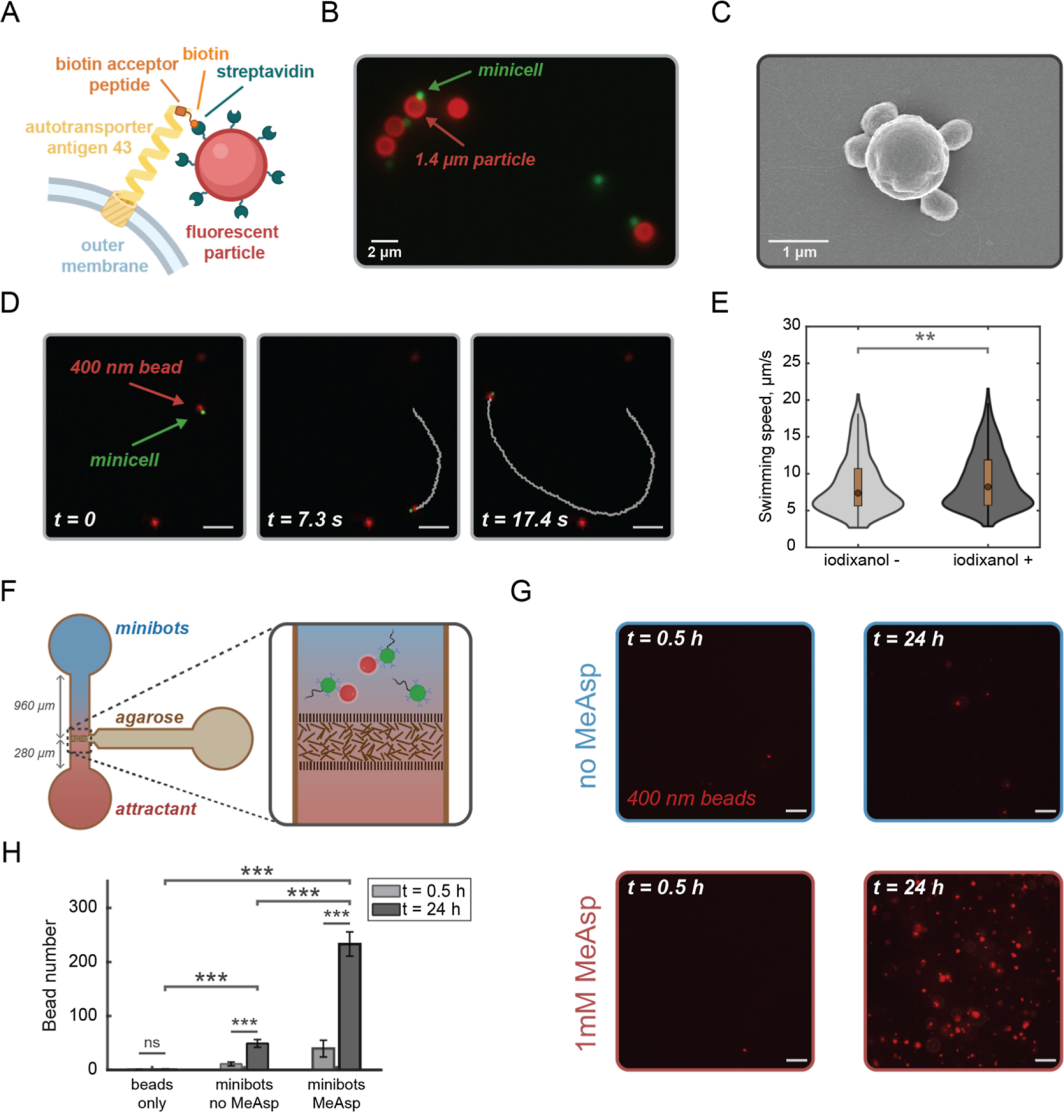
Motile and chemotactic minicell-based biohybrids enable cargo delivery toward a chemoattractant source. (A) A schematic of the attachment mechanism of streptavidin-coated fluorescent particles to the surface of minicells mediated by biotinylated autotransporter antigen 43 (Ag43). (B) Attachment of Δ*min*Δ*ycgR* + *flhDC* minicells (expressing GFP and modified Ag43 shown in green) to 1.4 μm fluorescent streptavidin-coated particles (red). The scale bar is 2 μm. (C) A scanning electron microscopy (SEM) image of a 1.4 μm streptavidin-coated particle with four attached minicells. The scale bar is 1 μm. (D) A time-lapse wide-field fluorescence series with a minicell (expressing GFP and modified Ag43, shown in green) carrying a 400 nm bead (shown in red) (see Movie S7). The scale bar is 5 μm; timestamps are shown. (E) Swimming speed of the minicells with attached 400 nm particles (minibots) in the absence and presence of 15% iodixanol. Total number of minibots included in the distributions: 450 (without iodixanol) and 1348 (with iodixanol). A two-sample *t*-test was used to calculate a significance value between the two conditions: *P* = 0.002 (**). (F) A schematic of the chemotactic chamber for the accumulation assays. Two wells are connected through a channel containing a porous agarose membrane; the lengths of both arms of the channel are shown. To establish a gradient, an attractant is added into one well while the minibots are loaded into the other one. Accumulation of fluorescent particles assessed by recording snapshots in the proximity of the attractant source (shown in the zoom-in panel). (G) Chemotactic accumulation of minibots in the presence of 1 mM MeAsp. The snapshots were taken after 30 min and 24 h after loading into the microfluidic device. The chip was kept at 22.5 °C during the entire experiment. The scale bar is 5 μm. (H) Quantification of the accumulated fluorescent particles performed with single particle counting. The particles attached to elongated parental cells, which accidentally appeared in the field of view (∼1%), were excluded from the quantification. The data represent the average across seven measurements per each condition for minibots and five measurements for the beads only control; the error bars are the standard deviations. A paired one-side *t*-test was used to calculate significance values for the measurements at *t* = 0.5 h and *t* = 24 h. A nonpaired *t*-test was used to compare the accumulation in the presence and in the absence of the attractant. Significance marks stand for: *P* ≤ 0.05 (*), *P* ≤ 0.01 (**), *P* ≤ 0.001 (***), *P* > 0.5 (ns).

We next explored whether minicells could transport particles of different sizes. Interestingly, while minicells were capable of actively exerting force onto 1.4 μm particles, these biohybrids were unable to swim along a persistent trajectory (Movies S5 and S6), presumably due to the instability of their geometrical configuration and unfavorable hydrodynamic effects. In contrast, minicells successfully transported particles approximately 400 nm in size, which are comparable to their own dimensions (Figure 5D). These “minibots” moved along persistent trajectories, demonstrating the effectiveness of the minicell-driven movement (Movie S7). Consistent with the slow dissociation rate of the streptavidin–biotin interaction^64^ and the expected formation of multiple biotin–streptavidin bonds between a minicell and a streptavidin-coated particle, no detachment of particles from minicells has been observed.

To further characterize the motility of minibots, we determined their swimming speed by tracking fluorescent particles rather than minicells themselves by applying a diffusion coefficient threshold to distinguish them from beads undergoing Brownian motion. The velocity of minibots varied widely, with an average value of 8.5 μm/s but reaching 15 μm/s for individual minibots (Figure 5E). The lower average speed in comparison to that of the unloaded minicells could be attributed to the increased load of attached particles. Furthermore, an unstable movement was observed for some minibots, likely due to the unbalanced geometrical configuration of a minicell with an attached particle. Additionally, we observed that both free particles and minibots sedimented over time, thereby limiting the observation time to several hours. We therefore increased the medium buoyancy and viscosity with the aim of mitigating sedimentation and stabilizing trajectories. Consistent with our expectations, the average speed of minibots increased in the presence of 15% (v/v) iodixanol up to 9.1 μm/s (Figure 5E). In combination with a similar effect observed for minicells in the presence of Ficoll (Figure 3D), these findings suggest that increasing viscosity might provide a general strategy for optimizing the motility of such “unstable” microswimmers, which could be attractive for biomedical applications that normally imply a higher viscosity of the surrounding environment.

Lastly, we investigated whether engineered motile minibots retained the ability to follow chemoattractant gradients. We first characterized the spatiotemporal dynamics of gradient formation in the microfluidic accumulation chamber, where an attractant continuously diffused through a porous membrane to form a stable linear gradient in the observation channel for at least 24 h (Figures 5F and S8). To qualitatively assess the chemotactic response of minibots, we then imaged their accumulation near an attractant source in the same microfluidic device, where MeAsp was continuously diffused through the membrane. To enhance the motility of minibots, 15% iodixanol was again added into the buffer. While no significant difference was observed between the number of minibots in the presence of the 1 mM MeAsp gradient source and in the buffer control half an hour after the loading, a significantly higher accumulation of minibots compared to the control was observed in the presence of MeAsp after 24 h of incubation (Figure 5G,H).

To estimate the efficiency of cargo delivery by minibots, we measured the passive diffusion of beads at the same concentration as was used for conjugation with minicells but without minibots (Figure 5H). After 24 h, fewer than one particle on average was detected in the observation channel, compared to approximately 49 beads delivered by minibots in the absence of an attractant and about 233 beads in the presence of MeAsp. This indicates that active delivery by motile minibots (without an attractant gradient) was roughly 80-fold higher than passive diffusion. Even more notably, the targeted delivery in the presence of MeAsp was five times greater than the untargeted delivery and nearly 400 times greater than passive diffusion of beads. These results demonstrate that the engineered motile and chemotactic minicell-based biohybrids can actively accumulate in response to localized chemoattractant release.

## Conclusions

Due to their submicron size, nonreplicating nature, high biocompatibility with hosts, feasibility of genetic engineering, and low-cost production, bacterial minicells have high potential as novel promising carriers for targeted drug delivery applications.^36–40,42,66,67^ The capacity of minicells to swim and follow concentration gradients could provide an opportunity for improving the specificity, penetration, and local accumulation of minicell-based drug carriers. In this study, we genetically engineered motile minicells with enhanced swimming ability and established an efficient protocol for their purification. These engineered minicells perform efficient chemotaxis, maintain their motility for several days, and show enhanced swimming at a higher viscosity. Finally, they can be used for the fabrication of motile minicell-driven biohybrids (minibots) that are capable of active cargo delivery toward an attractant source.

This opens new avenues for utilizing minicell-based biohybrids as a customized active cargo delivery platform with the potential of being fine-tuned for a specific biomedical application. A notable example is the use of motile minicell-based systems as a promising alternative to conventional cancer treatment strategies. Their small size and self-propulsion could facilitate their passage through leaky tumor vasculature and deeper penetration into poorly accessible tumor areas that are difficult to reach otherwise.^1,68^ By leveraging tumor-specific microenvironmental cues, such as hypoxia, acidic pH, and unique metabolic signatures,^68^ these systems can be engineered for chemotaxis-guided targeting and enhanced penetration, offering the potential to exceed the efficacy of passive delivery methods. This directional sensing of tumor-associated biochemical gradients, further combined with antibody-specific functionalization of the minicell surface,^37,38^ is likely to offer the most effective approach for precise targeting while minimizing off-target effects. Given the documented *in vivo* stability of minicells^37^ and the use of robust interactions, such as biotin–streptavidin, for minibot fabrication, their structural integrity under physiological conditions is encouraging, yet detailed validation is still required.

On the limitations side, the immunogenicity of bacterial components such as peptidoglycan, lipopolysaccharides, and flagella remains a critical consideration in the development of minicell-based drug delivery systems.^4^ Strategies to mitigate immune activation include generating minicells from commensal bacterial strains, applying camouflage coatings to the minicell surface, and employing less immunogenic flagellin variants. Alternatively, this inherent immunogenicity can be harnessed to stimulate antitumor immune responses, particularly within immunosuppressive tumor microenvironments.^8,42^ Lastly, establishing robust quality control measures and standardized scalable production protocols will be essential to ensure consistency, safety, and therapeutic efficacy for translation toward clinical and commercial applications.

## Experimental Methods

### Strain and Plasmid Construction

The derivatives of E. coli MG1655 were used in all conducted experiments. The entire *minCDE* operon (VS1869) and *fliC* gene (VS1953) were deleted using the λ Red recombination. *flgM* and *ycgR* knockouts in the Δ*min* background (VS1879, VS1894, and VS2050) were obtained with the P1 transduction protocol using MG1665Δ*flgM*::Kan^R^ and MG1665Δ*ycgR*::Kan^R^ as donor strains. In all strains, the kanamycin cassette was removed by FLP recombination and genetic modifications were verified by PCR.

To construct pTrc99a-*flhDC*, the pTrc99a backbone was digested with the *Xba*I/*Eco*RI restriction enzymes and ligated with the *flhDC* operon amplified from the E. coli MG1655 genome. pASM2-Ag43 was designed to allow for a high constitutive expression of biotinylated autotransporter antigen 43 (Ag43-BAP). Ag43-BAP was amplified from pOS233 ^17^ and inserted under the control of a strong constitutive promoter BBa_J23100 (https://parts.igem.org/Promoters/Catalog/Anderson). The plasmid pBAD-GFP was derived from pBAD33 and encoded the arabinose-inducible promoter P*BAD* controlling the expression of GFPmut2. pASM2-Ag43 and pBAD-GFP were constructed using Gibson assembly. All strains and plasmids, used in the study, are listed in Tables S1 and S2, respectively.

### Production and Purification of Motile Minicells

The protocol for minicell production and purification has been developed based on several previous studies^46–48^ and is illustrated in Figure 1A. The day before an experiment, an E. coli minicell-producing strain was grown overnight in TB at 37 °C. To generate minicells, the overnight cultures were diluted 1-in-350 in 20 mL of fresh TB medium and grown for 5 h in a shaking incubator (220 rpm) at 37 °C. To remove a large fraction of parental cells, the cultures were centrifuged in 50 mL falcons at 3220*g* and 4 °C for 15 min. The middle part of the supernatant was collected, mixed with a twice-larger volume of fresh TB, and further grown in a shaking incubator (at 220 rpm) at 37 °C for 1 h to restart the growth of the remaining parental cells. At the next step, rapidly dividing parental cells were targeted by 1 h ceftriaxone treatment (Sigma-Aldrich) in the final concentration of 250 μg/mL. The suspension was centrifuged in 50 mL falcons at 3220*g* at 4 °C for 15 min, and 5 mL, above the pellet, was collected. Minicells were finally concentrated by spinning at 10,000 rpm for 20 min. In case of strains carrying the pTrc99a-*flhDC* plasmid, the growth medium was supplemented with ampicillin (100 μg/mL).

### Motility and Chemotaxis Assays

The fraction of motile minicells, swimming speed, and chemotaxis drift were measured according to the protocols previously described in detail.^47,63^ In the motility assays, purified minicells were placed between two coverslips and sealed with vaseline. For the chemotactic drift measurements, minicells were loaded into two chambers of a polydimethylsiloxane (PDMS) microfluidic device filled with the motility buffer (6.15 mM K_2_HPO_4_, 3.85 mM KH_2_PO_4_, 100 μM EDTA, 67 mM NaCl, pH 7.0) supplemented with 1% (w/v) glucose, 0.01% (v/v) Tween 80, and either with or without 1 mM of a nonmetabolizable chemoattractant α-methyl-dl-aspartate (MeAsp).^62,63^ All motility and chemotaxis measurements were performed at 22.5 °C. Where indicated, the medium viscosity was adjusted using Ficoll based on the previously published data.^69^ Trajectories of single minicells were recorded in the middle of the channel between the chambers (chemotaxis assays) and in proximity to the bottom coverslip (motility assays) using phase-contrast microscopy (Nikon TI Eclipse, 40× objective NA 0.6, CMOS camera EoSens 4CXP) at an acquisition rate of 50 frames per second. To classify run and tumble events, the recorded trajectories were further analyzed with the custom ImageJ particle-tracking algorithm (https://github.com/croelmiyn/ParticleTracking). Swimming speed was computed across the population of motile minicells as the average instantaneous velocity within the classified runs. Chemotactic drift velocity *V*_*X*_ in the direction of an attractant gradient was calculated as the final displacement along each trajectory *i* normalized by its duration *T*_*i*_ as *V*_*X*,*i*_ = (*X*_end,*i*_ – *X*_start,*i*_)/*T*_*i*_ and averaged across all detected trajectories *V*_*X*_ = ∑*V*_*X*,*i*_ *T*_*i*_/∑*T*_*i*_. To account for the direct contribution of swimming speed into chemotactic drift velocity *V*_*X*_, chemotactic bias (*V*_*X*_/*V*) and chemotactic efficiency (*V*_*X*_/*V* ^2^) were additionally calculated.

For run duration analysis, runs shorter than 5 frames (0.1 s) were removed and the direction of each run *j* was calculated as $\alpha_{j}=\cos^{-1}\frac{\Delta y_{j}}{\sqrt{{\Delta x_{j}}^{2}+{\Delta y_{j}}^{2}}}$ , where Δ*x*_*j*_ = *x*_*j*,end_ – *x*_*j*,start_ and Δ*y*_*j*_ = *y*_*j*,end_ – *y*_*j*,start_. All runs with |α| < π/4 and 3π/4 < |α| < π were classified as up and down (the gradient), respectively (Figure 4A). The distributions of run durations for both subpopulations were plotted by using MATLAB.

Statistical analysis of motility and chemotactic drift measurements as well as the average run durations toward the attractant for different strains was performed with a two-sample *t*-test (ttest2, MATLAB). To compare run durations up and down the gradient, a paired one-side *t*-test has been applied (ttest, MATLAB). A *P*-value greater than 0.05 was considered as statistically insignificant.

### Flagellar Staining and Quantification

Carboxylic acid succinimidyl ester dye (Alexa Fluor 594 NHS ester from Molecular Probes) was used to stain flagella as previously described.^53^ Purified minicells were gently washed in the buffer (10 mM KPO_4_, 100 μM EDTA, 67 mM NaCl, 0.001% (v/v) Tween 80, pH 7.0), resuspended in 300 μL of the same buffer with pH 7.8 (pH was adjusted with sodium bicarbonate NaHCO_3_) and incubated with 50 μg of Alexa Fluor 594 in the dark at slow shaking (100 rpm) for 1.5 h. The labeled minicells were washed by gentle centrifugation in the buffer with pH 7.0 and imaged onto 2% (w/v) agar pads using a ZEISS Elyra 7 microscope in the laser widefield mode (63× NA 1.46 Oil objective, camera PCO Edge 4.2, excitation at 561 nm and emission at 595/50 nm).

### Flagellar Rotation Speed Measurements

To quantify flagella rotation speed, purified and labeled with Alexa Fluor 594 NHS ester dye, minicells were resuspended in the motility buffer (pH 7.0), supplemented with 1% (w/v) glucose and 10% (w/v) Ficoll 400, and placed in between two glass coverslips. Movies were recorded at 56 frames per second with the same imaging setup as for flagellar quantification under a controlled room temperature of 22.5 °C. To reduce the background signal from other minicells in the field of view, the area around a swimming minicell was manually selected (the examples are provided in Movies S3 and S4). The duration of all selected recordings was fixed to 128 frames. The PSD of the averaged fluorescence intensity within each 8 × 8 pixel square was calculated as a function of temporal frequency (ω/2π) using a one-dimensional fast Fourier transform algorithm, $PSD={\left|{\left\langle Ĩ\left(\omega \right)\right\rangle }_{8\times 8}\right|}^{2}$ , where *Ĩ*(ω) is the temporal Fourier transform of the pixel intensities. A custom ImageJ script *DFFM_1* is available at https://github.com/croelmiyn/FourierImageAnalysis. The raw PSD was corrected for Brownian motion as PSD/ω^2^. Since flagellar rotation is the only process that induces periodic changes of the pixel intensities, and the dominating peaks correspond to the frequency of flagellar rotation. These peaks were fitted by a parabolic function, and the frequency corresponding to the maximum was taken as a flagellar rotation frequency. The violin plots were generated using an open-source script (https://github.com/bastibe/Violinplot-Matlab). Statistical significance values were calculated with a two-sample *t*-test (ttest2) in MATLAB.

### Attachment of Streptavidin-Coated Particles to Minicells

In the particle attachment experiments, the minicell-producing Δ*min*Δ*ycgR* strain with pTrc99a-*flhDC* and pASM2-Ag43 plasmids was used. Cell cultures were grown overnight (14 h) in TB supplemented with ampicillin (100 μg/mL), kanamycin (50 μg/mL), and 2 μM biotin (Sigma-Aldrich) at 220 rpm at 37 °C. The cultures were collected next morning, and minicells were purified according to the procedure described above. In the case of minicells with the GFP reporter, the strain was additionally transformed with the pBAD-GFP plasmid, and chloramphenicol (30 μg/mL) and 0.01% (w/v) arabinose were added to the medium to induce GFP expression. Needed antibiotics, inducers, and biotin were added during all stages of the purification protocol.

The purified minicells were gently resuspended in 500 μL of the motility buffer (pH 7.0) supplemented with 0.5% (w/v) BSA and 1% (w/v) glucose. To conjugate them with particles, 5 μL of either 381 nm PolyAn Red4 or 1.4 μm PolyAn Red5 fluorescent PMMA streptavidin-coated particles (0.5% solids content, both purchased from PolyAn, Berlin) was added to the minicells and the mixture was incubated for 1 h at 20 °C (shaking at 160 rpm). To increase the efficiency of conjugation, the mixture was further kept overnight (12 h) at 4 °C. Next morning, minicells conjugated with the particles were placed in between glass coverslips and visualized with a ZEISS Elyra 7 microscope with a 40× NA 1.4 Oil objective, camera PCO Edge 4.2, and excitation laser line at 561 nm. Time-lapse movies were recorded at 27 frames per second. In the colocalization experiments, excitation laser lines at 488 and 561 nm were used, and the fluorescence was recorded with a dual camera PCO Edge 4.2 simultaneously in the GFP (523/55 nm) and mCherry (595/50 nm) channels. To reduce particle attachment to a glass surface, coverslips were rinsed with the motility buffer containing 0.5% (w/v) BSA prior to the loading of the sample. The motility measurements were performed at 22.5 °C. The swimming speed of the minibots was identified from the tracks of fluorescent particles in the mCherry channel. The particles, actively transported by minicells, were selected by setting a diffusion coefficient threshold that exceeds the Brownian motion of freely moving particles. The two-sample *t*-test was used to calculate *P*-value between two conditions.

### Scanning Electron Microscopy

Minicells, conjugated with streptavidin-coated particles were placed on silicon wafers. After settling for 30 min at room temperature, the samples were fixed with 2.5% (v/v) glutaraldehyde and incubated at 4 °C for half an hour. Next, dehydration was performed in gradually increasing concentrations of ethanol (25%, 50%, 75%, 90%, and 100%). After that, the samples were subjected to chemical drying with hexamethyldisilazane (HMDS) in ethanol at the concentrations: 33%, 50%, 67%, and 100% HMDS, and left to dry overnight in a fume hood. The next day, a 10 nm gold layer was sputtered onto the samples using a Leica EM ACE600 sputter coater, and the samples were visualized using a Zeiss Ultra 550 Gemini at an accelerating voltage of 3 keV with an in-lens detector.

### Chemotaxis Accumulation Assays

The chemotactic accumulation measurements of minicells with particles were performed in a microfluidic chamber with a porous membrane.^70^ The geometrical configuration of the chip is shown in Figure 5F. After the conjugation step, minibots were washed twice in the motility buffer (pH 7.0) supplemented with 0.5% (w/v) BSA and 1% (w/v) glucose at 6000 rpm for 7 min and gently resuspended in the same buffer mixed with 15% (v/v) iodixanol. When loading the microfluidic chip, 2% (w/v) low-gelling agarose, kept at 70 °C, was added into the middle well, allowing the porous membrane to solidify in the separation channel. Then, the motility buffer containing 0.5% (w/v) BSA and 15% iodixanol was quickly loaded into the side wells to equilibrate fluid pressure. After that, the concentrated mixture of minibots was loaded into the well with the longer channel, while either no or 1 mM MeAsp was added to the shorter one. The chip was covered with a glass coverslip to prevent evaporation and kept at 22.5 °C during the experiment. Fluorescent mCherry (595/50 nm) snapshots of the field of view close to the separation porous membrane were taken at 30 min and at 24 h after the loading. The beads attached to elongated parental cells, which accidentally appeared in the field of view (∼1%), were excluded from the quantification. A paired one-side *t*-test was used to calculate significance values for the measurements at *t* = 0.5 h and *t* = 24 h. A nonpaired *t*-test was used to compare the accumulation in the presence and in the absence of the attractant.

### CFU Assays

To measure the efficiency of ceftriaxone treatment, minicells were purified according to the protocol above with different ceftriaxone concentrations (0, 10 μg/mL, 35 μg/mL, 170 μg/mL, 330 μg/mL). The final mixture of purified minicells (100 μL) was then plated onto LB agar plates and incubated for 24 h at 37 °C. The next day, the colonies were counted. In the case of the absence of colonies, the plates were kept at 37 °C for one more day. Finally, colony-forming units were calculated per milliliter of the initial volume of the cultures, harvested for purification (20 mL).

## Supplementary Material





## Data Availability

All data needed to evaluate the conclusions in the paper are presented in the paper and the Supporting Information. Image analysis codes are available at https://github.com/croelmiyn/ParticleTracking and https://github.com/croelmiyn/FourierImageAnalysis.

## Notes

Additional experimental details and lists of strains and plasmids used in the study (PDF)
Swimming minicells derived from the original minicell-producing strain; swimming minicells derived from the engineered minicell-producing strain; a minicell derived from the original minicell-producing strain with labeled flagella; a minicell derived from the engineered minicell-producing strain with labeled flagella; a minicell actively rotating a 1.4 μm particle; two minicells applying a force onto a 1.4 μm particle; and a minicell actively transporting a 400 nm particle (ZIP)
