# Supplementary material for: Motile and Chemotactic Minicells and Minicell-Driven Biohybrids Engineered for Active Cargo Delivery
Source: ACS Appl Mater Interfaces. 2025 Jun 12;17(25):36387–99. doi: 10.1021/acsami.5c04638 (PMC12203475; doi:10.1021/acsami.5c04638)
Supplement: Supplementary file 1 [file am5c04638_si_001.pdf]

Supporting Information

Motile and Chemotactic Minicells and Minicell-Driven Biohybrids Engineered for Active Cargo Delivery

Irina Kalita[1], Remy Colin[1], Sarah Hoch[1], Saadet Fatma Baltaci[2,3], Metin Sitti[2,3,4], Victor Sourjik[1,*]

[1] Max Planck Institute for Terrestrial Microbiology and Center for Synthetic Microbiology (SYNMIKRO), Marburg, 35043, Germany

[2] Physical Intelligence Department, Max Planck Institute for Intelligent Systems, Stuttgart, 70569, Germany

[3] Stuttgart Center for Simulation Science, University of Stuttgart, Stuttgart, 70569, Germany

[4] School of Medicine and College of Engineering, Koç University, Istanbul, 34450, Turkey

[*] Corresponding author: victor.sourjik@mpi-marburg.mpg.de

This PDF file includes:

- Figures (S1 to S8)
- Tables (S1 to S2)
- Legends for the Movies (S1 to S7)

Other Supplementary Materials for this manuscript include:

- Movies S1 to S7

Supplementary Figures

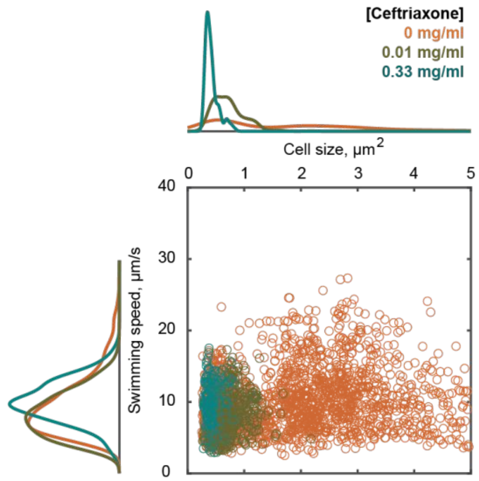

Figure S1: Enrichment for minicell subpopulation with ceftriaxone treatment. Single-cell correlation between swimming speed and cell size in the absence, with intermediate (10 μg/ml) or high (330 μg/ml) concentration of ceftriaxone used during the purification procedure (Figure 1A). The marginal distributions of swimming speed and cell size are plotted on the sides. *E. coli* MG1655 Δ*minCDE* strain was used for minicell production.

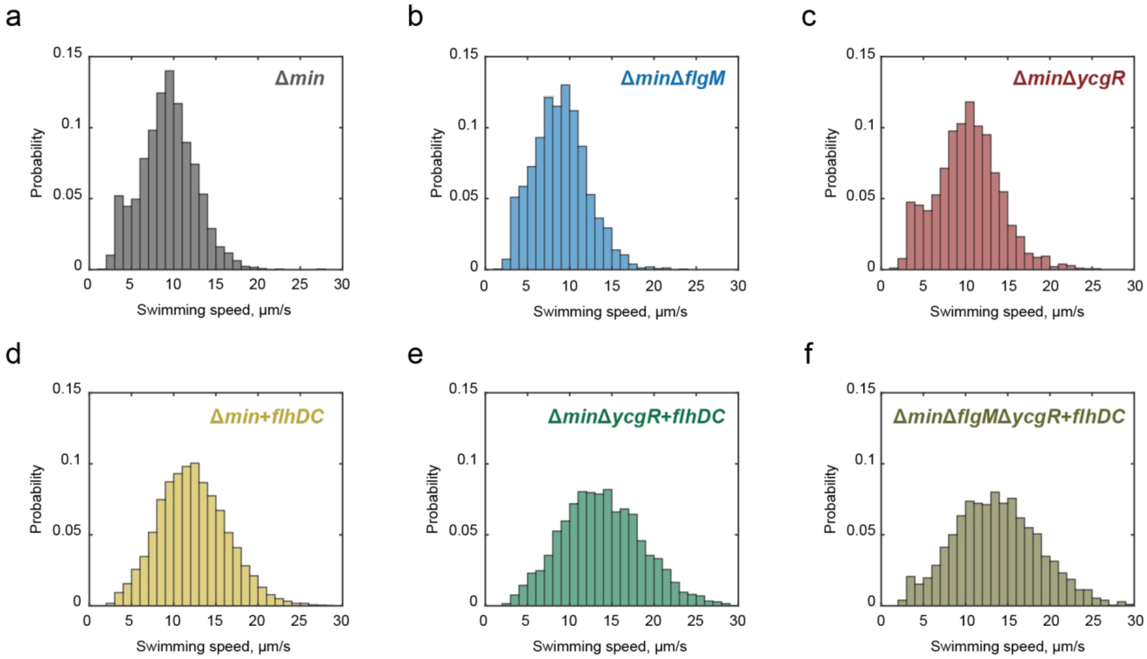

Figure S2: Swimming speed distributions of minicells produced by all constructed strains. Histograms of swimming speed of the purified minicells generated by Δ*min* (**a**), Δ*min*Δ*flgM* (**b**), Δ*min*Δ*ycgR* (**c**), Δ*min* + *flhDC* (**d**), Δ*min*Δ*ycgR* + *flhDC* (**e**), and Δ*min*Δ*flgM*Δ*ycgR* + *flhDC* (**f**) strains. Swimming speed was calculated for each detected trajectory with single-cell tracking analysis. The data from all replicated experiments were combined. The number of minicells included in the distributions: 2557 (Δ*min*), 2591 (Δ*min*Δ*flgM*), 4650 (Δ*min*Δ*ycgR*), 3975 (Δ*min* + *flhDC*), 5743 (Δ*min*Δ*ycgR* + *flhDC*), 2035 (Δ*min*Δ*flgM*Δ*ycgR* + *flhDC*).

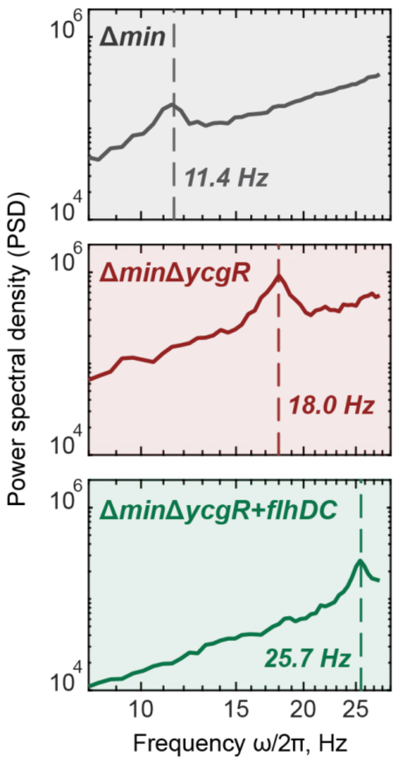

Figure S3: Identification of flagellar rotation frequency for individual minicells produced by Δ*min*, Δ*min*Δ*ycgR*, and Δ*min*Δ*ycgR*+*flhDC* strains. Examples of power spectral densities (PSDs) computed from time-lapse recordings of minicells with labelled flagella swimming in the motility buffer supplemented with 1 % glucose and 10 % Ficoll 400. Examples of analyzed movies for the minicells, produced by Δ*min* and Δ*min*Δ*ycgR*+*flhDC* strains, are provided in Supporting Movie S3 & S4, respectively. PSDs of the averaged fluorescence intensity within each 8×8 pixel square were calculated as a function of temporal frequency ($\omega/2\pi$) using a one-dimensional fast Fourier transform algorithm and corrected for Brownian motion (Experimental Methods). The dominating peaks correspond to the frequency of flagellar rotation as the only process with periodic changes. These peaks were fitted by a parabolic function and the frequency corresponding to its maximum was taken as a flagellar rotation frequency (dashed vertical lines).

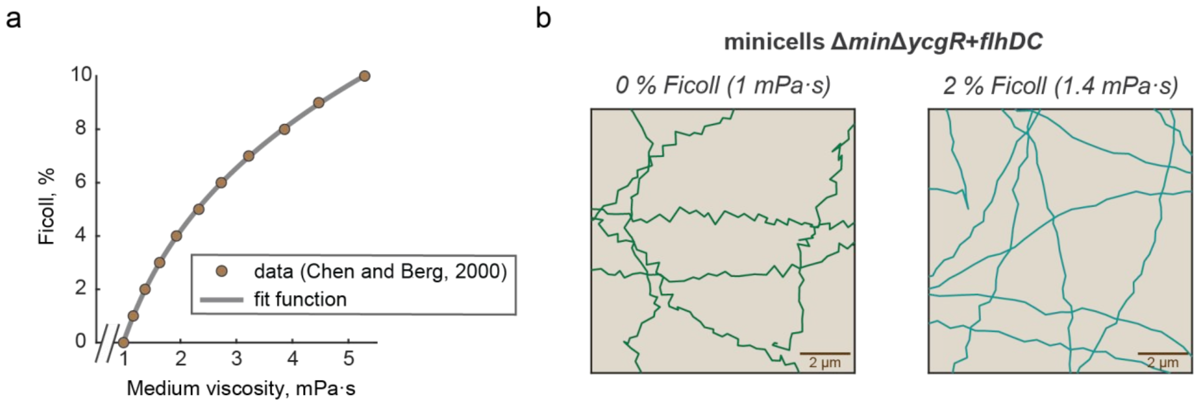

Figure S4: Swimming behavior of the improved minicells, produced by Δ*min*Δ*ycgR* + *flhDC* strain, in media with different viscosity. (a) Calibration of medium viscosity for different percentages of Ficoll**.** Viscosity of the motility buffer supplemented with different percentages of Ficoll 400 (w/v) calculated in mPa·s. The data for calibration was adapted with permission from Chen X.; Berg H.C. *Biophys J.* **2000**, 78(2): 1036-41. Copyright 2000 The Biophysical Society. Published by Elsevier Inc. The data for 22.7 °C was fitted by a polynomial function in the range from 0 to 10 % of Ficoll. **(b)** Representative examples of trajectories of the minicells produced by Δ*min*Δ*ycgR* + *flhDC* strain in the motility buffer with 0 % and 2 % Ficoll. The scale bars are 2 µm.

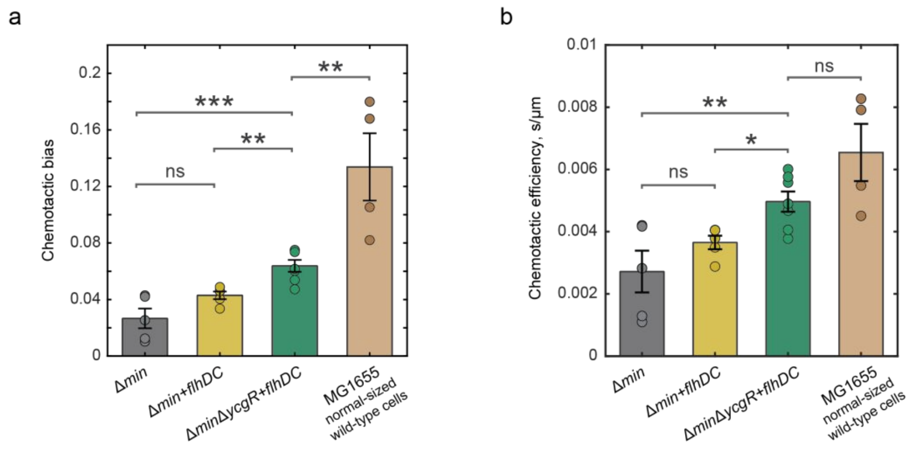

Figure S5: Chemotactic bias and chemotactic efficiency of minicells and normal-sized wild-type cells in the presence of MeAsp gradient. (a) Chemotactic bias, defined as chemotactic drift normalized by the swimming speed, for the minicells produced by Δ*min*, Δ*min+flhDC* and Δ*min*Δ*ycgR+flhDC* strains or for the wild-type *E. coli* MG1655 cells in the presence of 0-1 mM MeAsp gradient in the device shown in Figure 4A. **(b)** The efficiency of chemotactic response of the minicells produced by Δ*min*, Δ*min+flhDC*, and Δ*min*Δ*ycgR+flhDC* strains or of the wild-type *E. coli* MG1655 cells. Chemotactic efficiency was calculated as chemotactic bias divided by swimming speed. In **(a)** and **(b)**: each data point represents the average chemotactic bias (or chemotactic efficiency) for a population of purified minicells while the bars show the mean among the averages of at least four replicated experiments. The error bars are standard error of the mean. Two-sample *t*-test was used to calculate significance values: $P \leq 0.05$ (*), $P \leq 0.01$ (**), $P \leq 0.001$ (***), $P > 0.5$ (ns).

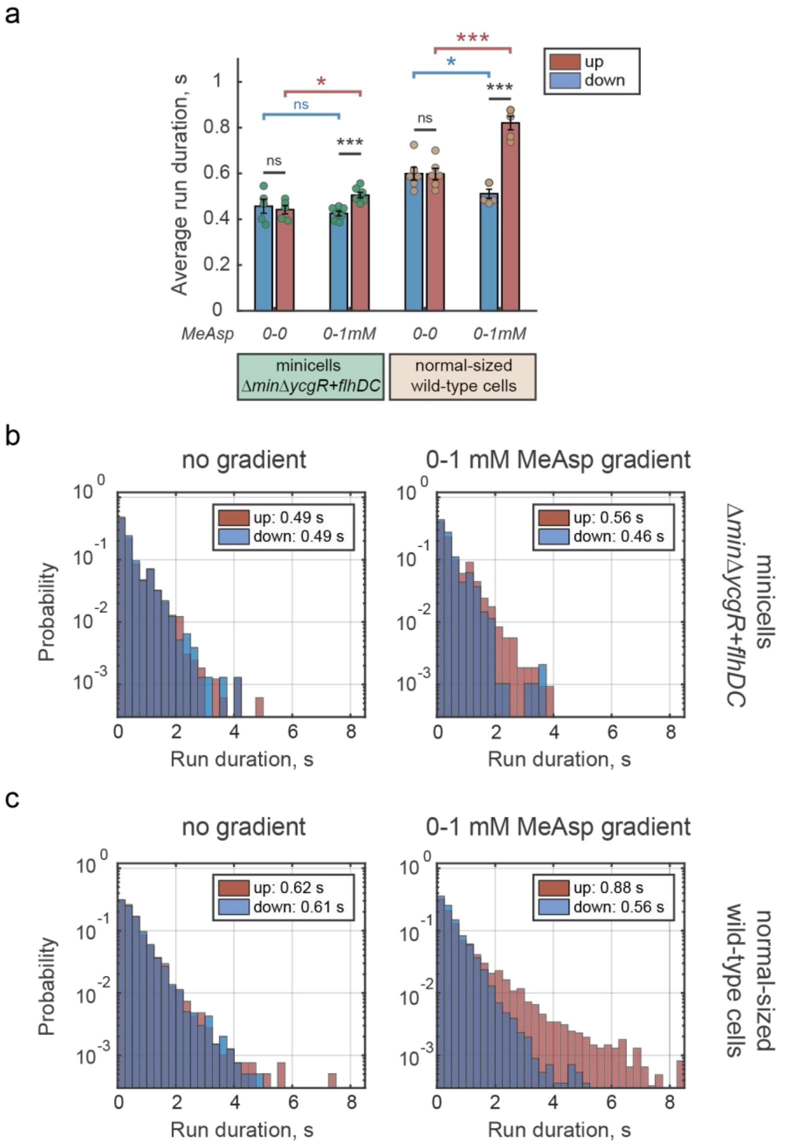

Figure S6: Run durations in the absence and in the presence of MeAsp gradient for minicells and wild-type cells. (**a**) Average run duration in the buffer and in the presence of MeAsp gradient for the Δ*min*Δ*ycgR* + *flhDC* minicells and normal wild-type MG1655 cells. The average values were calculated for at least four replicated experiments for each strain. The error bars are standard errors of the mean. To compare run durations up and down the gradient, paired one-side t-test has been applied. Non-paired *t*-test was used to compare run durations up (or down) in the absence and in the presence of the gradient. Significance marks stand for: $P \le 0.05$ (*), $P \le 0.01$ (**), $P \le 0.001$ (***), $P > 0.5$ (ns). (**b, c**) Histograms of run durations in the motility buffer and in the presence of 0-1 mM MeAsp gradient for a representative dataset with the Δ*min*Δ*ycgR* + *flhDC* minicells (**b**) and normal-sized MG1655 wild-type cells (**c**). The runs shorter than 5 frames (0.1 s) were excluded from the analysis; all other runs were separated based on their directionality: up or down the chemoattractant gradient (Figure 4A). The average run durations in both directions for the exemplified dataset are shown in the corresponding legend. The number of analyzed trajectories in (**b**): 2488 (no gradient) and 1739 (in the presence of the gradient); and in (**c**): 4254 (no gradient) and 5147 (in the presence of the gradient).

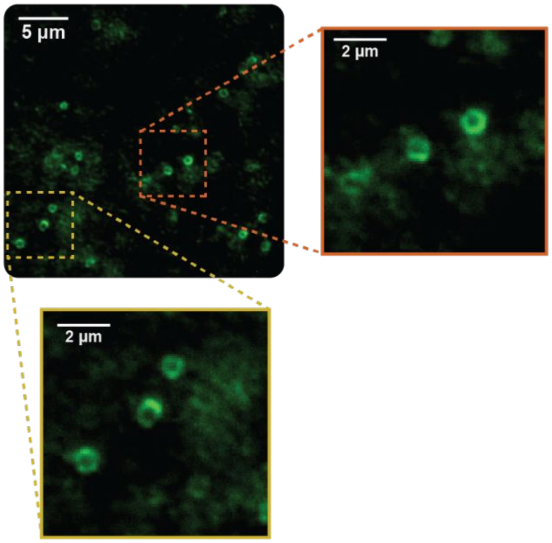

Figure S7: Expression and membrane localization of the biotinylated autotransporter Ag43 in minicells. A wide-field fluorescence microscopy image of minicells expressing modified Ag43 on the surface and labelled with NeutrAvidin dye. Scale bar is 5 µm in the large image and 2 µm in the zoom-in images.

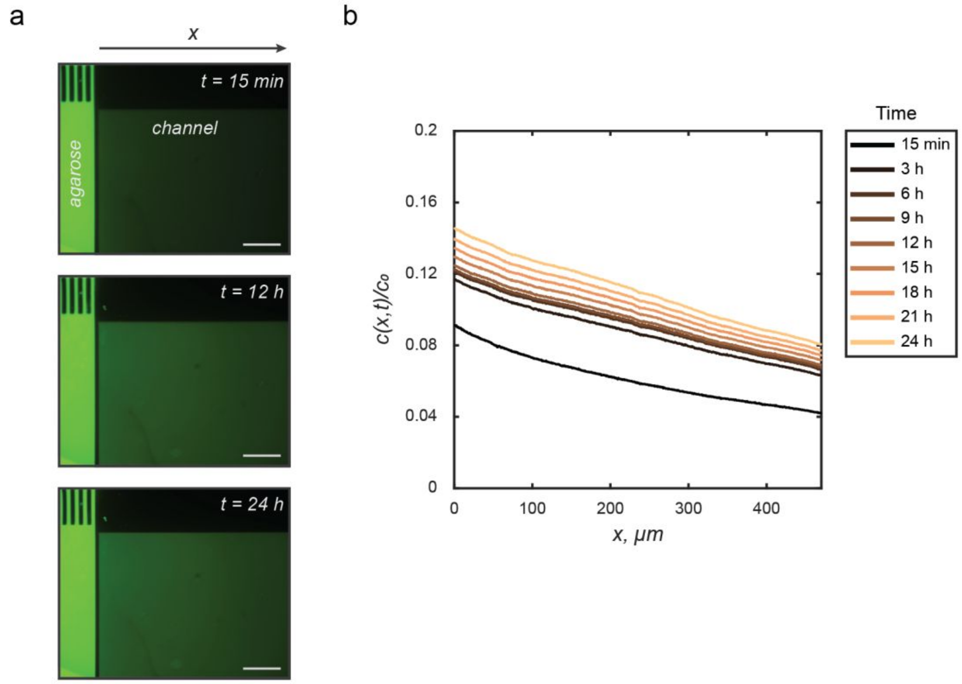

Figure S8: Spatiotemporal characterization of a gradient in the microfluidic accumulation assays. (**a**) Representative examples of the observation channel (top view) separated by a porous agarose membrane from the source of an attractant in the accumulation chamber. The position of a porous agarose membrane is indicated. The gradient is visualized with fluorescein (shown in green), a fluorescent dye with the diffusion coefficient similar to that of MeAsp. The snapshots of the observation channel with fluorescein are shown at 15 min, 12 hours, and 24 hours after the loading. Scale bars are 100 µm. (**b**) The intensity profiles of fluorescein concentration along x-axis over a 24-hour experiment. The intensity profile $c(x,t)$ was normalized by the intensity in the source reservoir c_0.

Supplementary Tables

Table S1: *Escherichia coli* strains used in the study.

Strain	Genotype	Source or reference
MG1655	*F- lambda- ilvG- rfb-50 rph-1*	[*]
VS1869	*F- lambda- ilvG- rfb-50 rph-1 ΔminCDE*	This work
VS1879	*F- lambda- ilvG- rfb-50 rph-1 ΔminCDE ΔflgM*	This work
VS1894	*F- lambda- ilvG- rfb-50 rph-1 ΔminCDE ΔycgR*	This work
VS1953	*F- lambda- ilvG- rfb-50 rph-1 ΔminCDE ΔfliC*	This work
VS2050	*F- lambda- ilvG- rfb-50 rph-1 ΔminCDE ΔycgR ΔflgM*	This work

* Blattner F.R.; Plunkett G. III; Bloch C.A.; Perna N.T.; Burland V.; Riley M.; Collado-Vides J.; Glasner J.D.; Rode C.K.; Mayhew G.F.; Gregor J.; Davis N.W.; Kirkpatrick H.A.; Goeden M.A.; Rose D.J.; Mau B.; Shao Y. The complete genome sequence of *Escherichia coli* K-12. *Science* **1997**, *277* (5331), 1453-62. doi: 10.1126/science.277.5331.1453.

Table S2: Plasmids used in the study.

Plasmids	Description	Source
pTrc99-*flhDC*	pTrc99-derivative carrying *flhDC* operon under control of IPTG-inducible promoter	This work
pASM2-Ag43	A plasmid used for expression of biotinylated version of the autotransporter Ag43-BAP under control of a strong constitutive promoter	This work
pBAD-GFP	pBAD33-derivative carrying GFPmut2 gene under control of arabinose-inducible promoter	This work

Supplementary Movies

Movie S1: Swimming minicells derived from the original minicell-producing strain.

Motile minicells produced by the original Δ*min* strain and purified with the described protocol. Scale bar is 10 μm. The movie is displayed in real time.

Movie S2: Swimming minicells derived from the engineered minicell-producing strain.

Motile minicells produced by the engineered Δ*min*Δ*ycgR* + *flhDC* strain and purified with the described protocol. Scale bar is 10 μm. The movie is displayed in real time.

Movie S3: A minicell derived from the original minicell-producing strain with labelled flagella.

An example of a single minicell generated by the original Δ*min* strain and labelled with Alexa Fluor 594 carboxylic acid succinimidyl ester dye. Scale bar is 2 μm. The movie is displayed in half-real time.

Movie S4: A minicell derived from the engineered minicell-producing strain with labelled flagella.

An example of a single minicell generated by the engineered Δ*min*Δ*ycgR* + *flhDC* strain and labelled with Alexa Fluor 594 carboxylic acid succinimidyl ester dye. Scale bar is 2 μm. The movie is displayed in half-real time.

Movie S5: A minicell actively rotating a 1.4 μm particle.

A bright-field video with a minicell, produced by the engineered Δ*min*Δ*ycgR* + *flhDC* strain expressing the biotinylated version of antigen 43, actively rotating a 1.4 μm streptavidin-coated microparticle. Scale bar is 2 μm. The movie is displayed in 2X real time.

Movie S6: Two minicells applying a force onto a 1.4 μm particle.

A wide-field fluorescence video with two minicells (in green), produced by the engineered Δ*min*Δ*ycgR* + *flhDC* strain carrying GFP and modified Ag43 expression plasmids, actively applying a force onto a fluorescent streptavidin-coated 1.4 μm microparticle (in red). Scale bar is 2 μm. The movie is displayed in half-real time.

Movie S7: A minicell actively transporting a 400 nm particle.

A wide-field fluorescence video with a minicells (in green), produced by the engineered Δ*min*Δ*ycgR* + *flhDC* strain expressing GFP and biotinylated Ag43, carrying a fluorescent streptavidin-coated 400 nm bead (in red). Scale bar is 5 μm. The movie is displayed in real time.
